# Carbon Orientation in the Diatom *Phaeodactylum tricornutum*: The Effects of Carbon Limitation and Photon Flux Density

**DOI:** 10.3389/fpls.2019.00471

**Published:** 2019-04-16

**Authors:** Parisa Heydarizadeh, Brigitte Veidl, Bing Huang, Ewa Lukomska, Gaëtane Wielgosz-Collin, Aurélie Couzinet-Mossion, Gaël Bougaran, Justine Marchand, Benoît Schoefs

**Affiliations:** ^1^Metabolism, Bioengineering of Microalgal Molecules and Applications, Mer Molécule Santé, Le Mans University, IUML FR 3473 CNRS, Le Mans, France; ^2^Physiology and Biotechnology of Algae Laboratory, IFREMER, Nantes, France; ^3^Mer Molécule Santé, University of Nantes, IUML FR 3473 CNRS, Nantes, France

**Keywords:** **:** diatom, carbon deficiency, carbon metabolism, stress, light intensity, regulation, biotechnology, phospho*enol*pyruvate

## Abstract

Diatoms adapt to changing environmental conditions in very efficient ways. Among the mechanisms that can be activated, the reorientation of carbon metabolism is crucial because it allows the storage of energy into energy-dense molecules, typically lipids. Beside their roles in physiology, lipids are commercially interesting compounds. Therefore studies dealing with this topic are relevant for both basic and applied science. Although the molecular mechanisms involved in the reorientation of carbon metabolism as a response to a deficiency in nutrients such as nitrogen or phosphorus has been partially elucidated, the impacts of carbon availability on the implementation of the reorientation mechanisms remain unclear. Indeed, it has not been determined if the same types of mechanisms are activated under carbon and other nutrient deficiencies or limitations. The first aim of this work was to get insights into the physiological, biological and molecular processes triggered by progressive carbon starvation in the model diatom *Phaeodactylum tricornutum*. The second aim was to investigate the effects of the growth light intensity on these processes. For such a purpose three different photon flux densities 30, 300, and 1000 μmol photons m^-2^ s^-1^ were used. The results presented here demonstrate that under carbon limitation, diatom cells still reorient carbon metabolism toward either phospho*enol*pyruvate or pyruvate, which serves as a hub for the production of more complex molecules. The distribution of carbon atoms between the different pathways was partially affected by the growth photon flux density because low light (LL) provides conditions for the accumulation of chrysolaminarin, while medium light mostly stimulated lipid synthesis. A significant increase in the amount of proteins was observed under high light (HL).

## Introduction

Diatoms constitute the most abundant group of marine eukaryotic organisms with more than 200 genera and approximately 100,000 species and most have still to be discovered (e.g., Heydarizadeh et al., 2014; Bork et al., 2015; Vinayak et al., 2015; Beauger et al., 2019). It is well established that diatoms are able to adapt to a broad range of environmental conditions including light irradiances and nutrient concentrations through adjustment of their physiology and biochemical activity (e.g., Schoefs et al., 2017) while maintaining high growth rates and a high efficiency of carbon incorporation into different organic metabolites (Nymark et al., 2009). Yet, excessive or insufficient incident photon flux density constrains diatom optimal performance in terms of biomass and metabolite composition (Goldman, 1980; Vidoudez and Pohnert, 2008; Barofsky et al., 2009, 2010; Carvalho et al., 2011). These metabolites are generated along a network of biochemical pathways, the core of which being occupied by the central carbon metabolism. Many stress conditions result in the reorientation of the carbon metabolism toward the accumulation of energy-dense molecules such as lipids (for reviews, see Sayanova et al., 2017; Zulu et al., 2018). The accumulation of these energy-dense molecules is only possible when a source of carbon is available (Parupudi et al., 2016; Heydarizadeh et al., 2017). The fact that most of the stress conditions trigger the same type of response let us hypothesize that the reorientation of carbon metabolism toward the accumulation of energy-dense molecules could constitute a default response mechanism of diatoms to a stress. At first glance, testing this hypothesis seems important only from the academic point of view but a more careful examination of the question reveals its broad interest. In natural conditions, the weak CO_2_ solubilization in water forces algae to use carbon concentration mechanism(s) (CCM) to acquire more carbon, the amount of which being still limiting for biomass production. On the other hand, our calculations predict that even with 100% utilization of industrial CO_2_ waste for lipid production, there is not enough atmospheric CO_2_ to be converted for feeding all transport now (Vinayak et al., 2015). Also, the costs of CO_2_ used in industrial setups are prohibitive. Altogether, it seems that CO_2_-limitation could be a limiting factor for the development of an efficient algal biotechnological process as it is already for algae living in the natural environment.

The metabolic reorientation of carbon can be tightly regulated by light (Dron et al., 2012). Recently, Heydarizadeh et al. (2017) showed that under carbon-limited conditions, a sudden transition in the growth light intensity impacts the use of pyruvate/phospho*enol*pyruvate for the production of building blocks: organic acids under low light (LL) and lipids and proteins under high light (HL). Little is still known about carbon flux direction inside the cell, partition between the different pathways, transporters and molecular mechanisms behind light acclimation in diatoms (e.g., Marchand et al., 2018). One way to follow these mechanisms consists in exploring genes encoding proteins associated with the pathways because the level of gene expression may affect enzyme amount and, thus, flux distribution (Depauw et al., 2012; Heydarizadeh et al., 2014).

The experiments presented here have been performed on clonal populations of the marine diatom *Phaeodactylum tricornutum* because the completion of its genome sequencing (Bowler et al., 2008) made it a “model” diatom for genomic, biochemical and physiological studies (Thiriet-Rupert et al., 2016; Gruber and Kroth, 2017; Kroth et al., 2017, 2018).

## Materials and Methods

### Experimental Strategy, Growth Rate, and Sampling

Pt4-type *Phaeodactylum tricornutum* Bohlin (UTEX 646) was grown according to Heydarizadeh et al. (2017). Approximately 10^5^ cells mL^-1^ of an axenic culture in exponential phase of *P. tricornutum* were batch cultured in 200 mL of f/2 prepared with artificial seawater (Guillard and Ryther, 1962) in three biological replications. The growth medium was supplemented with NaHCO_3_ (8%) at the initiation of the culture. The growth photon flux densities of 30, 300, and 1000 μmol photons PAR m^-2^ s^-1^ were used as low light (LL), optimal (ML) and high (HL) photon flux densities, respectively, using cool-white fluorescent tubes (Philips Master TL-D 90 DE luxe 58W/965 and Osram L58/77 FLUORA). These levels of irradiance were chosen according to the light saturation index value (E_k_) parameters obtained for *P. tricornutum* grown under 300 μmol photons PAR m^-2^ s^-1^ (Heydarizadeh et al., 2017).

Cell counting was carried out regularly using a Neubauer hemocytometer. Growth rate was obtained after fitting growth kinetics with the sigmoid equation using the software CurveExpert Basic^1^. For short time intervals, the daily division rate (μ_ddr_) was estimated using (Equation 1):

$${\text{μ}}_{ddr}(\mathrm{cell}\;{\text{d}}^{-1})\;=\;[\ln\;{\text{N}}_{t}\;-\;\ln\;{\text{N}}_{t-1}]/\text{Δ}t\;\;\;\;\;\;\;\;\;\;\;\;\;\;\;\;\;\;\;\;\;(1)$$

where N_t_ and N_t-1_ are the number of cells at time t and t-1.

Because cell physiology is dependent on the growth conditions, the elucidation of the underlying mechanisms requires us to compare samples that are in closely similar physiological states (Heydarizadeh et al., 2014). To fulfill this condition, the time-course of the daily division rate (μ_ddr_) (Equation 1) along the growth period was calculated for each photon flux density (data not shown). Regardless of the light intensity, the time-courses present a bell-shape curve peaking at the middle of the exponential phase and zeroing in lag and plateau phases (data not shown). Sampling times were chosen when the time-course of μ_ddr_ was reaching its maximum or was close to zero, defining the three sampling times used for each lighting condition. Three biological replicates were conducted.

The photon flux densities were measured using a 4π waterproof light probe (Walz, Germany) connected to a Li-Cor 189 quantum meter. In all cases a 12/12 h light/dark cycle and 21°C conditions were maintained.

### Pigment Extraction, Photosynthetic, and Respiratory Activity, PI-Curve

Rates of oxygen evolution were measured at 21°C in the light and in the dark using a fiber-optic oxygen meter (Pyroscience^®^ FireSting O_2_, Germany) with a diatom suspension (1.5 mL). The cells were illuminated at their growth photon flux density. Respiration was measured in the dark immediately after each measurement of photosynthesis. Gross photosynthesis was calculated as net photosynthesis plus respiration, assuming that respiratory activity (R_d_) was the same in light and in darkness, respectively (Heydarizadeh et al., 2017). Because, R_d_ is impacted by light photon flux density used during the gross photosynthesis measurement, R_d_ was measured after the oxygen evolution measurement in the light. Calculated values were normalized to cell density or chlorophyll (Chl) *a* amount. Chl *a*, Chl c and total carotenoids were measured according to Heydarizadeh et al. (2017).

### Chlorophyll Fluorescence Yield Measurement

Chlorophyll fluorescence yield was monitored at the growth temperature using a fluorimeter FMS-1 (Hansatech^®^) using 2 mL of culture according to Roháček et al. (2014). To avoid CO_2_ shortage during measurements, the cultures were provided with NaHCO_3_ (final: 4 mM, 0.2 M/stock).

The analysis of the qN relaxation kinetics into its components qNi, qNf, and qNs was performed as explained in Roháček et al. (2014). The quality of the regression procedure was assessed using two parameters: (1) values of qN_1_ = qNf + qNi + qNs were compared to the experimental values of qN and (2) coefficient of determination of the fit (R^2^) was taken as a measure of how well observed outcomes are replicated by the regression model (Steel and Torrie, 1960). In this work a regression was considered as good when *R*^2^ ≥ 0.90.

### Quantification of Intracellular Carbon and Nitrogen, Cellular Carbon, and Nitrogen Quotas, C and N Uptake Rate

*Q_N_* and *Q_C_* were determined for each growth phase using a C/N elemental analyzer (EAGER 300, Thermo Scientific). Samples were filtered through precombusted Whatman GF/C glass filters under gentle vacuum (50 mm Hg) and dried at 70°C for 48 h. The volume of solution filtered was adjusted to have either 0.1 or 0.3 10^8^ cells per filter.

The C (ρ_C_) and N uptake rates (ρ_N_) were estimated according to Marchetti et al. (2012) using Equation 2 and Equation 3 when cultures were at equilibrium (i.e., at maximum growth rate and at stationary phase). During phase 1, *ρ* was also estimated by *μ∗ Q*, which is a default value for *ρ*. Indeed, since cultures were inoculated 3 days before sampling for *Q* assessment, they were considered to be near equilibrium.

$${\text{ρ}}_{C}(\mathrm{pg}\;{\mathrm{cell}}^{-1}{\text{d}}^{-1})\;=\;{\text{μ}}_{ddr}Q_{C}\;\;\;\;\;\;\;\;\;\;\;\;\;\;\;\mathrm{(2)}$$

$${\text{ρ}}_{N}(\mathrm{pg}\;{\mathrm{cell}}^{-1}{\text{d}}^{-1})\;=\;{\text{μ}}_{ddr}Q_{N}\;\;\;\;\;\;\;\;\;\;\;\;\;\;\;(3)$$

$$\mathrm{Total}\;\mathrm{amount}\;\mathrm{of}\;\text{C}\;\mathrm{immobilized}\;(\mathrm{pg})\;=\;Q_{C}{\text{N}}_{phase\_3}\;\;\;\;\;\;\;\;\;\;\;\;\;\;\;\;\;\;\;\;\mathrm{(4)}$$

$$\mathrm{Total}\;\mathrm{amount}\;\mathrm{of}\;\text{N}\;\mathrm{immobilized}\;(\mathrm{pg})\;=\;Q_{N}{\text{N}}_{phase\_3}\;\;\;\;\;\;\;\;\;\;\;\;\;\;\;\;\;\;\;(5)$$

where N_phase_3_ represents the number of cells in phase 3.

### Determination of the Protein Content

To isolate total proteins, cell cultures were centrifuged (5000 × *g*, 5 min, 4°C), overlaid in 2 mM EDTA (ethylenediaminetetraacetic acid) and homogenized using Ultra Turrax^®^ (IKA-Analysentechnik GmbH) for 10 min. After centrifugation of the mixture (13,000 × *g*, 5 min, 4°C), protein amount in the supernatant was measured according to Bradford (1976).

### Determination of the Lipid Content

Lipids were extracted from 10^8^ to 10^9^ cells following a modified Bligh and Dyer method (Bligh and Dyer, 1959). Briefly, freeze-dried cells were steeped in dichloromethane/methanol (2:1,v/v) for 2 h at room temperature. The extract was filtered, washed with distilled water and evaporated to dryness under a stream of nitrogen. The lipids were determined gravimetrically.

### Determination of Chrysolaminarin Content

Cellular β-1,3-glucan was extracted according to Granum and Myklestad (2002) with some modifications. Briefly, 10^7^ cells were harvested by filtration and each filter was transferred directly to a glass vial and stored at -20°C until analysis. The cellular β-1,3-glucans were extracted by H_2_SO_4_ (50 mM) at 60°C for 10 min using a water bath. The extract was centrifuged at maximum acceleration for 10 min (4°C). The resulting supernatant was collected and transferred into a new tube and dried at 60°C. Twenty five μL of 3% aqueous phenol and 2.5 mL concentrated H_2_SO_4_ were added to 2 mL of sample and the mixture was immediately vortexed. The tubes were allowed to stand for 30 min, and then cooled with running water. Absorbance at 485 nm was measured. The amount of chrysolaminarin was calculated using glucose (stock concentration: 50 μg mL^-1^) as a standard.

### Primer Design

A total of 33 different enzymes involved in carbon metabolism pathways of the diatom *P. tricornutum* were selected and the corresponding genes (74) coding each enzyme (see Figure 6 and Supplemental Data 1) were searched in genomic data published by Kroth et al. (2008).

### RNA Extraction and qRT-PCR

1.5 10^8^ cells were collected by filtration at the 3 phases and at the 3 light intensities. Total RNA extraction, reverse transcription and real time PCR reactions were performed using the primers and the protocol described in Heydarizadeh et al. (2017). Out of the 12 housekeeping gene (HKG) candidates, the most stable (tbp, ubi, and rps) were selected to normalize the target genes (TG). The calculation of the relative expression (RE) was based on the comparative Ct method (Livak and Schmittgen, 2001): RE = ((E_TG_) ^ΔCt TG^)/(E_HKG_) ^ΔCt HKG^) with ΔCt = Ct_Calibrator_- Ct_sample_ (Pfaffl et al., 2004). Three biological replicates were used. Heatmap analyses were performed using Netwalker 1.0 (Komurov et al., 2012).

## Results

### Effect of Light Intensity on the Growth of *Phaeodactylum tricornutum*

**Figure 1:**
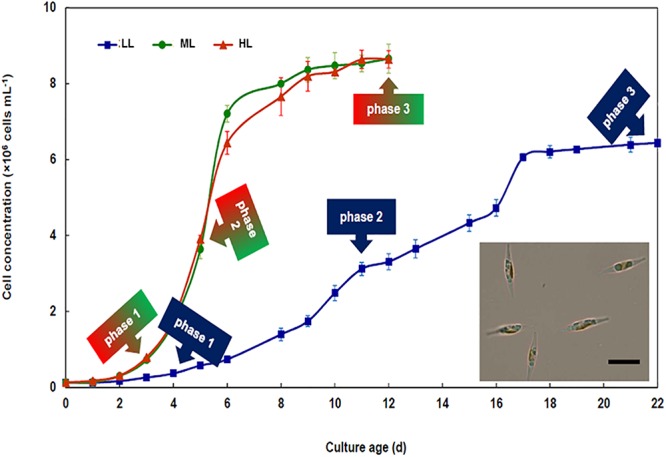
Growth of *Phaeodactylum tricornutum* under different light intensities. Time course of cell density of cultures developing under 30 (LL), 300 (ML), and 1000 (HL) μmol photons PAR m^-2^ s^-1^. Each curve presents typical growth phases, i.e., lag (phase 1), exponential (phase 2), and plateau (phase 3). The sampling time in each phase is indicated using arrows. The insert presents a light microscopy picture of *Phaeodactylum tricornutum* grown under ML. The bar indicates 10 μm. Mean values obtained from three biological replicates and SD are presented.

Regardless the growth irradiance intensity, the growth curve of *P. tricornutum* could be fitted using a logistic law. The curves presented typical phases, i.e., lag, exponential and plateau phases. In the rest of the manuscript, we will refer to these different phases as phase 1, phase 2, and phase 3, respectively (Figure 1). No significant difference (*p* < 0.05) between the growth curves under ML and HL was observed. Cell density under LL was significantly lower (*p* < 0.05) than under ML and HL and growth phases were delayed in LL. Accordingly, the specific maximum growth rate (μ) and generation time (G), were similar under ML and HL, whereas these values were lower under LL (Table 1).

**Table 1:** Impact of the light intensity on culture growth rate and generation time of *Phaeodactylum tricornutum*.

Irradiance (μmol	30 (LL)	300 (ML)	1000 (HL)
photons PAR m^-2^ s^-1^)
μ (d**^-^**^1^)	0.340 ± 0.003^∗^	0.873 ± 0.009	0.865 ± 0.010
G (d)	2.039 ± 0.048^∗^	0.797 ± 0.008	0.806 ± 0.009

*Growth rates (μ) and generation times (G) were calculated from the curves of Figure 1 using the equation indicated in the “Materials and Methods” section. Under LL, the rate of cell division was circa 30% less than under ML and HL. Mean values ± SE (n = 3–5). Significant different data are indicated by an asterisk (Tukey Test, p ≤ 0.05)*.

### Pigment Content

**Figure 2:**
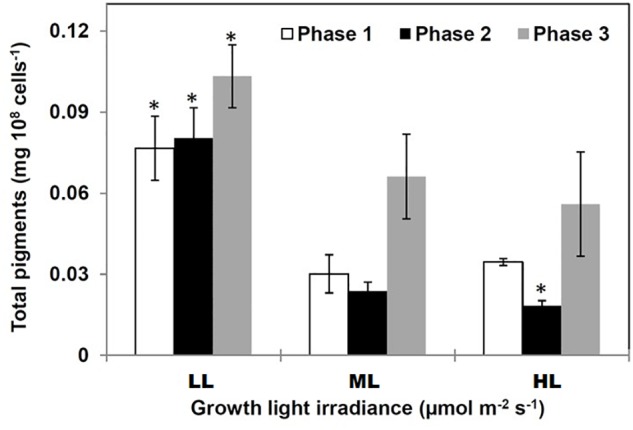
Total pigments in *Phaeodactylum tricornutum* grown under different light intensities. Pigment accumulated in diatoms in two growing conditions: (1) under LL (regardless the growth phase) and in phase 3 (regardless the growth photon flux density). Data are mean values ± SE (*n* = 3) and error bars represent SD. Means followed by asterisks are significantly different from the corresponding value in ML (*p* < 0.05, student’s *t*-test).

Total pigment content was higher under LL compared to ML and HL regardless the growth phase. The total pigment content increased along with growth (Figure 2) due to the accumulation of Chl *a* and total carotenoids (Supplemental Data 2). However, the pigment content in phase 2 was impacted by the light intensity, i.e., it decreased when the photon flux density increased (Figure 2). The Chl a/Chl c ratio varied in opposite directions under LL and ML/HL: under LL, it increased from phases 1 to 2 and then decreased until phase 3 (Supplemental Figure SD2.1). At the end of phase 3 the ratio was similar for all conditions.

### Photosynthetic and Respiratory Activities

**Figure 3:**
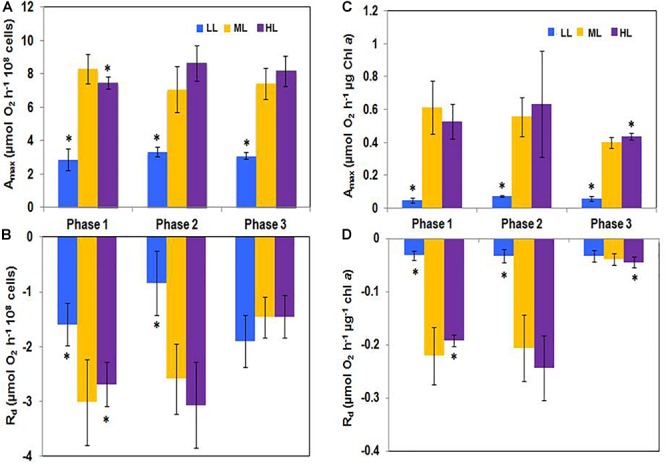
**(A,C)** Net photosynthesis (A_max_) and **(B,D)** respiration (R_d_) activities in *Phaeodactylum tricornutum* grown under different light intensities. A_max_ and R_d_ were significantly higher under ML and HL than under LL. However, their values did not change along the growth at any of the grown photon flux densities. Under LL, A_max_ was reduced while respiration was high, staying, however below. Values represent the mean ± SE (*n* = 3) and error bars represent SD. Means followed by asterisks are significantly different from the corresponding value in ML (*p* < 0.05).

Regardless if the net photosynthesis (A_max_) and respiratory activity (R_d_) were expressed relatively to the cell number (Figure 3A,B) or to the Chl *a* content (Figure 3C,D), A_max_ was always higher than R_d_. When taken individually, R_d_ and A_max_ were relatively constant during growth but R_d_ decreased in phase 3 suggesting a strong reduction of the metabolic activity.

### Photochemical and Nonphotochemical Quenching Analysis

**Figure 4:**
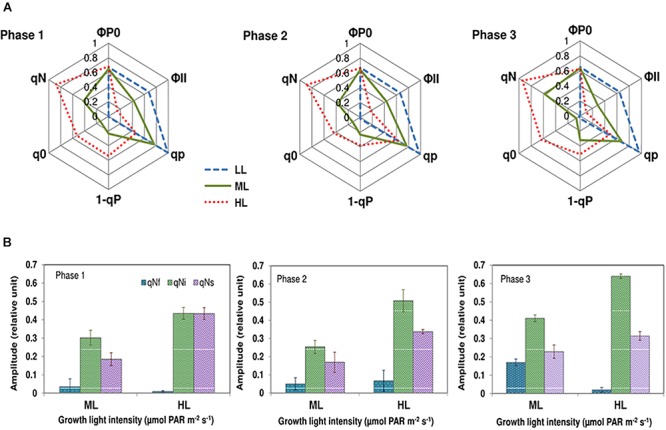
Chlorophyll fluorescence kinetic parameters during the induction and the relaxation of the nonphotochemical quenching in *Phaeodactylum tricornutum* grown under different light intensities and during different growth phases. **(A)** The fluorescence kinetic parameters during the induction of the nonphotochemical quenching. The maximum quantum yield of PSII photochemistry (ΦP0) in cells grown under different light condition and different growth phases was constant (almost 0.6), suggesting that cells were healthy. This result contrasts with the effective quantum yield of photochemical energy conversion in PSII (ΦP0), which progressively reduced from LL to HL. The photochemical quenching (qP) that quantifies the actual fraction of PSII reaction centers staying open during the illumination. qP values decreased as the light intensity increased in an antiparallel manner with 1-qP, that quantifies the fraction of closed reaction centers. The nonphotochemical quenching parameters qN and q0 reflect the excess radiation converted to heat during the actinic radiation. Under LL, qN, and q0 are close to zero, indicating that under this lighting condition, there was no excess of absorbed energy. The intensity of these parameters was higher under ML and HL, suggesting that under these photon flux densities, part of the incoming energy needed to be dissipated as heat. **(B)** The components qNf, qNi, and qNs during the relaxation of the nonphotochemical quenching. Light-adapted cells relaxed qN in the dark (Supplemental Data 3). The mathematical analyses of the relaxation kinetic allowed the determination of three individual components that are denoted qNs, qNi, and qNf. The intensity of qNi and qNs were always the largest in cells grown under HL whereas the intensity of qNf was the largest under ML, except in phase 2 in which the values were not significantly different. The proportion of qNi increased from phases 1 to 3. qNs was the second mostly intense components. Its amplitude decreased from phases 1 to 3 under HL while it remained constant under ML.

Typical Chl *a* fluorescence recordings are presented in Supplemental Figure SD3.1. Figure 4A compares the variations of photochemical and nonphotochemical processes using characteristic parameters (photochemical: ΦP0, ΦII, qP, 1-qP; nonphotochemical: (q_0_, qN)) during growth under the different light intensities. The meaning of the parameters and the equations used for calculations are presented in Supplemental Table SD3.1. The maximum quantum yield of photosystem II (PSII) photochemistry (ΦP0) in cells grown under different light conditions and different growth phases was around 0.6 (Figure 4A) suggesting that cells were healthy. This result contrasts with the effective quantum yield of photochemical energy conversion in PSII (ΦII), which progressively reduced from LL to HL. When compared to ML, the effective quantum yield of photochemical energy conversion in PSII was higher, about 60, 53, and 125% (Phases 1, 2, and 3, respectively) in cells grown under LL. No significant change of qP was observed during the different growth phases under either of the light intensities showing that diatoms were well adapted to the growth conditions. However, the qP values decreased as the light intensity increased. Under HL, qP values were approximately 50% of the values under LL (Figure 4A). The values of 1-qP, that quantifies the fraction of closed reaction centers, varied accordingly (Figure 4A). The absorption of an excess of photons triggers nonphotochemical quenching mechanisms of energy dissipation as heat (Roháček et al., 2008). The related parameters qN and q0 reflect the excess radiation converted to heat during the actinic radiation. These parameters increased with increasing photon flux density. Under LL, they were close to zero, indicating that under this lighting condition, there was no excess of absorbed energy. To decipher the mechanisms contributing to the dissipation of the excess of energy activated under ML and HL, relaxation of the nonphotochemical quenching was recorded. When adapted to light conditions (Fs phase) cells were placed in the dark, qN gradually relaxed (Supplemental Figure SD3.1). In agreement to Roháček et al. (2014) three individual components were found: qNi relies on the dissipation of the proton gradient (ΔpH relaxation) and diatoxanthin epoxidation. qNf seems to be related to fast conformational changes occurring within the thylakoid membranes in the vicinity of the PSII complexes, whereas qNs could be related to photoinhibition and/or partial dissipation of the pH gradient (for a detailed explanation, see Supplemental Data 3). Regardless the growth phase and the growth light, the less intense component was qNf while the most intense component was qNi. The amplitude of qNf remains at the basic levels except in phase 3 under ML. The intensity of qNi and qNs were always higher in cells grown under HL than under ML whereas the intensity of qNf was always higher in cells grown under ML than under HL, except in phase 2 in which the values were not significantly different from each other. The proportion of qNi increased from phases 1 to 3 (Figure 4B). qNs was the second mostly intense component. Its amplitude slightly decreased from phases 1 to 3 under HL while it remained constant under ML (Figure 4B).

### N and C Fluxes to Lipids, Carbohydrates, and Proteins

**Figure 5:**
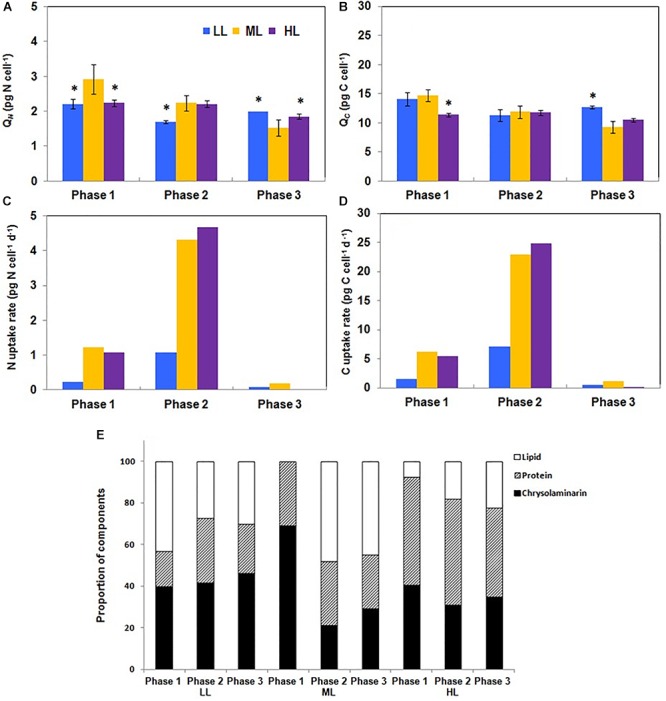
Modifications of the carbon, nitrogen, chrysolaminarin, protein and lipid content of *Phaeodactylum tricornutum* during growth under different photon flux densities. **(A,B)** The carbon and nitrogen cellular quota are not constant along growth. Under LL, *Q_N_*, and *Q_C_* decreased from phases 1 to 2 and increased from phases 2 to 3 whereas under ML, *Q_N_*, and *Q_C_* decreased continuously. Under HL, the decrease occurred only between phases 2 and 3. **(C,D)** The carbon and nitrogen uptake are maximum in phase 2. The rate of C and N uptake were the most intense in phase 2 and very reduced in phases 1 and 3. **(E)** The celluar quota in proteins, lipids and chrysolaminarin is impacted by the growth photon flux density. The relative amount of LPC was greatly impacted by the photon flux density. Lipids were the most abundant in phase 1 under LL whereas under ML and HL, they were barely detectable **(E).** Under LL, the relative abundance of lipids decreased during the transition from phases 1 to 2 and then remained constant until phase 3. This contrasts with ML and HL for which lipid proportion increased until phase 2 (ML) or phase 3 (HL). Data are mean values ± SE (*n* = 3) and error bars represent SD. Means followed by asterisks are significantly different from the corresponding value in ML (*P* ≤ 0.05, student’s *t*-test).

Because primary metabolism and physiological activities mostly rely on the C and N availability and cell uptake, the cellular N and C quota (*Q_N_* and *Q_C_*, respectively) were recorded. The time-courses of *Q_N_* and *Q_C_* were different according to the irradiance level: under LL, *Q_N_* and *Q_C_* decreased from phases 1 to 2 and increased from phases 2 to 3. Under ML, *Q_N_* and *Q_C_* decreased continuously whereas under HL, the decrease occurred only between phases 2 and 3 (Figure 5A,B). N and C uptake rates were the most intense in phase 2 and very reduced in phase 3 (Figure 5C,D). When expressed relatively to *Q_N_*, *Q_C_* varied only significantly in phase 3 in function of the growth light intensity: it increased from LL to HL (data not shown). When normalized to the Chl amount, the *Q_C_*/Chl *a* between phases 1 and 2 changed according to the growth light intensity: under LL, it slightly decreased, remained constant under ML and increased in HL. In phase 3, the values were similarly weak for each condition (data not shown).

The yield of C fixation ranged between 73 (LL and ML) and 78% (HL) whereas the yield of N fixation was around 10%, regardless of the light intensity (Table 2). The fixed N and C are used for the synthesis of cellular building blocks including lipids, proteins and chrysolaminarin (collectively “LPC”). To evaluate if a shift in C and N orientation occurred, the total amount of LPC was measured in the culture media and in the cells. None of these compounds could be detected in the culture media (data not shown), showing that export of such material was low or under the detection limits. The relative amount of LPC was greatly impacted by light intensity. For instance, in phase 1 and under LL, lipids represent a bit more than 40% of the total cellular mass of LPC whereas under ML and HL, lipids were barely detectable at that stage of growth (Figure 5E). Under LL, the relative abundance of lipids decreased during the transition from phases 1 to 2 and then remained constant until phase 3. This contrasts with ML and HL for which the lipid proportion increased until phase 2 (ML) or phase 3 (HL) (Figure 5E).

**Table 2:** Quantitative and relative amounts of C and N immobilized in the cells in phase 3.

	LL	ML	HL
Total C amount (mg)	197	197	199
Relative amount of the initial C consumed (%)	73	73	78
Total N amount (mg)	3.0	2.3	2.8
Relative amount of the initial N consumed (%)	10	8	9

*At the end of the studied period, approximately the same proportion of the initial carbon content has been consumed*.

The relative abundance of chrysolaminarin was the highest (70%) in phase 1 under ML. It dramatically decreased during the transition to phase 2 whereas under LL and HL it did not change by more than 8%. The relative amount of proteins was the highest (55%) under HL. Under this growth irradiance, it decreased only from phases 2 to 3 by *circa* 10% (Figure 5E).

### Changes in Selected mRNA Expression During Growth

**Figure 6:**
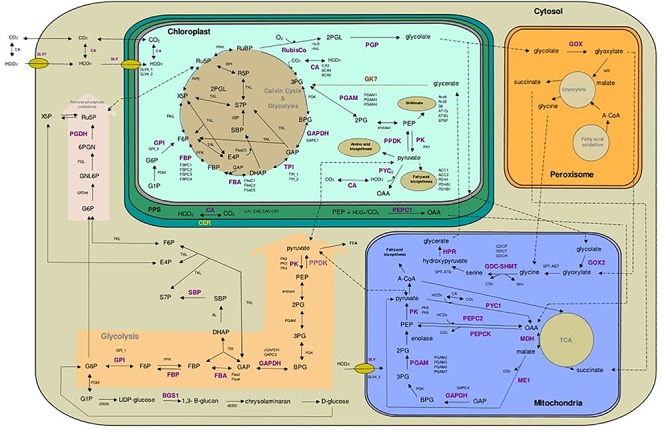
Cellular pathways and processes of central carbon metabolism in *Phaeodactylum tricornutum*. All red depicted enzymes are the key enzymes and their related gene/isogenes are listed beside. RUBP, D-ribulose-1,5-bisphosphate; 3PG, 3-phospho-*D*-glycerate; BPG, 1,3-diphosphateglycerate; GAP, D-glyceraldehyde-3-phosphate; DHAP, dihydroxyacetone phosphate; FBAC, chloroplastic fructose-1,6-bisphosphate aldolase; FBP, fructose-1,6-bisphosphate; F6P, D-fructose-6-phosphate; X5P, D-xylulose-5-phosphate; Ru5P, D-ribulose-5-phosphate; SBP, D-sedoheptulose-1,7-bisphosphate; S7P, D-sedoheptulose-7-phosphate; R5P, D-ribose-5-phosphate; E4P, D-erythrose-4-phosphate; 2PGL, 2-phosphoglycolate; 2PG, 2-phosphoglycerate; PEP, phospho*enol*pyruvate; OAA, oxaloacetate; A-CoA, acetyl-CoA; G1P, D-glucose 1-phosphate; G6P, D-glucose-6-phosphate; GNL6P, D-glucono-δ-lactone-6-phosphate; 6PGN, 6-phospho-D-gluconate; PPS, periplasmic space; ER, endoplasmic reticulum (chloroplastic); ACC1, ACC2, Acetyl-CoA-carboxylase; PDH1, PDHA1, PDHB, pyruvate dehydrogenase E1; BGS1, glycosyl transferase.

**Figure 7:**
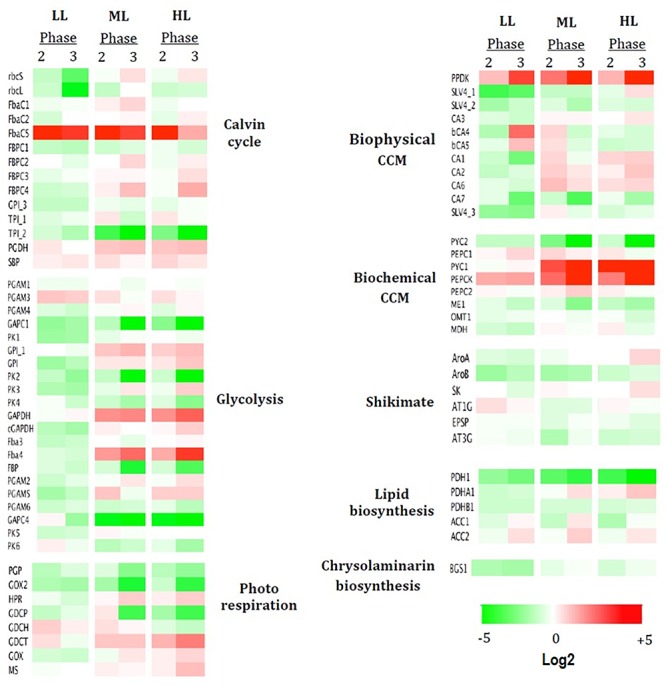
mRNA profiles of genes either up- or down-regulated during three growth phases under three light conditions. Are presented the log2 expression pattern of 74 genes related to carbon metabolic pathways, with the greatest differences in expression (red: high, green: low). Columns represent growth photon flux densities under the different growth phase, phase 1 being taken as reference. Heatmap performed using Netwalker 1.0. Dysregulated heatmap showing the expression pattern of 74 genes related to carbon metabolic pathways, with the greatest differences in expression (red: high, green: low). Columns represent three growth phases under low light (LL) and high light (HL). Heatmap performed using Netwalker 1.0.

To elucidate the reorganization of the metabolic circuits, the expression levels of 74 genes coding 33 enzymes involved in the central carbon metabolism were studied. A detailed description of the carbon metabolism is presented in Figure 6. A global view (heatmap) of the changes in the expression of the 74 genes of *P. tricornutum* based on growth phases under the three light intensities is shown in Figure 7. Out of the 74 genes, only 12 showed a particular profile regardless the light intensity (Figure 7): a particularly high up-regulation for PEPCK, FbaC5 and PPDK is observed while down-regulation occurred for PGP, GOX2, PYC2, GAPC1, PK2, CA7, TPI_2, ME1, and PDH1) (Figure 7). Under LL, genes encoding the HCO_3_^-^ transporters SLV4_1 and SLV4_3 involved in the biophysical CCM were down-regulated (up to -10 and -3-fold their expression in phase 1, respectively) while the two carbonic anhydrases (CA) (bCA4 and bCA5) were highly up-regulated in phase 3 (+12- and +3.5-fold the expression of these genes in phase 1). In the same conditions, the plastid rbcL and rbcS encoding two subunits of the RuBisCO from the Calvin cycle were down-regulated, (respectively, -11 and -3-fold the expression of phase 1 for phase 3). Most of the genes coding enzymes of the glycolysis in the three cell compartments in which glycolysis occurs were particularly down expressed. The genes encoding PGAM3 and GDCH (+3.5 and +4-fold respectively, in phase 2 compared to phase 1) were, however, up-regulated (Figure 7).

The pattern of mRNA expression during the growth of the culture for ML and HL was quite similar (Figure 7). An up-regulation of the genes encoding PYC1, Fba4, GDCT, GPI_1, GAPDH, and FBPC4 was highlighted (Figure 7) though not observed in LL. Genes particularly down-regulated along the growth in ML and HL (while not observed in LL) include genes encoding: (1) the single mitochondrial GAPC4 involved in glycolysis, reaching for ML (-29 and –47–fold, respectively, in phases 2 and 3 the expression of this gene in phase 1) and for HL (-27 and –61–fold, respectively); (2) FBP and PK4 both involved in cytosolic glycolysis (Figure 6).

### Light Intensity Influenced mRNA Expression

**Figure 8:**
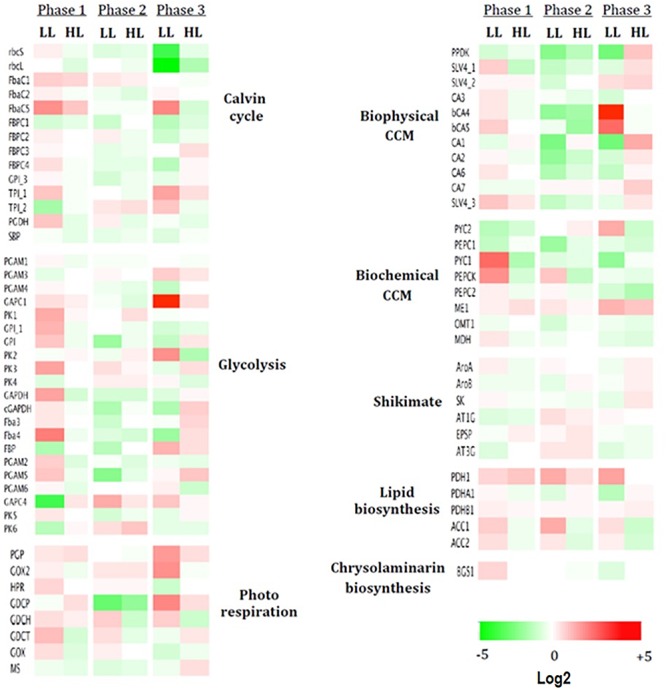
Dysregulated heatmap of up- or down-regulated genes under different light intensities. Are presented the log2 expression pattern of 74 genes related to carbon metabolic pathways, with the greatest differences in expression (red: high, green: low). Columns represent three growth phases under low light (LL) and high light (HL). Heatmap performed using Netwalker 1.0.

To determine how gene expression can be regulated under different light intensities, mRNA levels in each growth phase were compared and log_2_ fold change expressed relatively to ML (Figure 8). The strongest differences are found for LL and in particular in phases 1 and 3.

In phase 1, LL stimulated the expression of most of the genes studied and in particular (1) most of the genes encoding enzymes of the Calvin cycle and glycolysis and (2) mitochondrial genes encoding PYC1 and PEPCK from the biochemical CCM (+11 and +7-fold, respectively, their expression in ML). No sharp differences are observed for HL (Figure 8).

In phase 2, the strongest differences in LL and HL compared to ML were found in the biophysical CCM where mostly down-regulations were observed (Figure 8).

In phase 3, particular pathways are up-regulated: (1) the biophysical CCmml: mostly genes encoding the two bCAs (bCA4 and bCA5) with +36 and +13-fold, respectively, in LL while mostly genes coding aCA and HCO_3_^-^ transporters (+4.5-fold in CA1) in HL (Figure 8); (2) the photorespiration pathway in LL: +8.5, +7, and +6-fold for, respectively, the mitochondrial GDCP, GOX2, and PGP (Figure 7) and (3) several genes encoding enzymes of the Calvin cycle (+8 and +5-fold for FbaC5 and TPI_1 respectively) or the chloroplast glycolysis (+60-fold for GAPC1). The genes encoding the plastid rbcS and rbcL were, however, strongly down-regulated in LL (-7.5 and -11-fold, respectively).

## Discussion

Diatoms are among the most important contributors to the 100 gigatons of CO_2_ yearly converted into biomaterials and organic compounds (Field et al., 1998). Both the capacity of carbon fixation and the fate of fixed carbon atoms are strongly impacted by environmental factors. Among them, carbon availability (Heydarizadeh et al., 2017) and light (Darko et al., 2014) occupy unique places because the former provides the atom units required for the production of other cellular molecules whereas the latter is a vehicle for environmental information and of energy for photosynthesis, that in turn, is used for the synthesis of carbon based molecules. Diatoms acclimate to changing light intensities in a very efficient way (Wagner et al., 2006; Roháček et al., 2014). The regulation mechanisms involved in this process are still not completely elucidated (Nymark et al., 2009; Bailleul et al., 2010; Chauton et al., 2013). In this study, we have compared the impacts of the growth photon flux densities on the reorientation of the carbon metabolism of *P. tricornutum* grown under progressive CO_2_ limitation with the aim to highlight how the partitioning of carbon among its potential sinks is impacted.

### The Photon Flux Density Impacts Growth and the Energetic Metabolism

The effects of environmental factors on cell development, physiology and regulation mechanisms depend on cell status (Jia et al., 2015). Therefore, a careful study on the effects of growth light intensity requires the comparison of cells from cultures at similar developmental stages. To fulfill this requirement, the actual growth rate was calculated and the samples were prepared when the actual growth rate was at either maximum or minimum. Under LL, the mitosis frequency was *circa* three times lower than under ML or HL, indicating that the low abundance of photons constituted a limiting factor for growth of *P. tricornutum*. Our values are lower than those reported for *P. tricornutum* grown under the same nominal irradiances (Geider et al., 1985) due to the use of different light sensors (planar (2π) in Geider et al. (1985) versus spherical (4π) in this study). Both studies, however, agree on the higher growth rate under ML. When compared to ML, the additional photons brought by HL did not promote growth rate but generated a stress as suggested by the increase of the different mechanisms of excess energy dissipation. Altogether, these results agreed with those already published on this topic (e.g., Xiang et al., 2015).

The time-course of Chl *a* and total carotenoids accumulations under ML and HL differed significantly from those of LL grown cells, affecting the Chl a/Chl c ratio, a proxy for the size of the light harvesting antenna complex (Lamote et al., 2003; Nguyen-Deroche et al., 2012). Under LL, the ratio increased from phases 1 to 2 and then decreased until phase 3 indicating progressive LHC enlargement. At the end of phase 3, the antenna size was similar regardless the light intensity. In phase 3, under ML and HL, R_d_, A_max_ and rETR were reduced, suggesting a strong reduction of the metabolic activities. Under LL, R_d_ increased relatively to A_max_ suggesting the increase of the requirement of energy from nonphotosynthetic machinery.

### The Carbon Availability Along the Growth Indicates a Carbon Limitation

Among nutrient elements, C and N, above all else, are absolutely required for growth and the synthesis of metabolites. The variation of C- and N-uptake rates followed the rule observed by Marchetti et al. (2012) with *Isochrysis affinis galbana*, i.e., uptake variations follow those of growth rate. The C consumption was elevated along growth as over 70% had been consumed when the culture reached phase 3. Actually, it was estimated that only 4.5 μmol NaHCO_3_/10^6^ cells remained in phase 3. This level is far lower than that sufficient for optimal cell doubling (Riebesell et al., 1993). Altogether, this reasoning suggests that C deficiency was mostly responsible for the stationary phase. The fact that under ML and HL, the Chl *a* and total carotenoids amounts were the highest in phase 3 suggested that the cellular shading effect might also be partly responsible for the occurrence of the stationary phase. Alternatively, this phenomenon could also be interpreted as a way to enhance the production of ATP and NADPH through the photochemical reactions of photosynthesis for carbon fixation by RuBisCO. This change was less intense under LL probably because the pigment cellular quota was already almost at its maximum. The slight increase in the qN values observed in phase 3 suggests that energy production and energy expenditure were somehow more unbalanced than during phase 2. This imbalance was deeper in HL and to a lesser extent in ML conditions because the rETR was strongly reduced, providing good conditions for ROS production and photoinhibition. The photosynthetically fixed C was mostly used to synthesize lipids, proteins and carbohydrates. It was reported that increasing the irradiance level triggered an increase of the *Qc* of the diatom *Thalassiosira pseudonana* (Taraldsvik and Myklestad, 2000; Shi et al., 2015). Our measurements did not confirm this conclusion. The discrepancy may result from progressive exhaustion of the carbon source. It is interesting to note that, in phase 2, the pH of the medium (8.7) is more alkaline than in phase 1 (8.2). In *Skeletonema costatum*, such an increase favors CO_2_ uptake and the accumulation of amino acids (Taraldsvik and Myklestad, 2000). In our conditions, *P. tricornutum* accumulated relatively more proteins under HL.

As in cyanobacteria and green algae, diatoms possess CCMs for up-taking dissolved inorganic carbon from the surrounding environment. Two types of CCM have been reported to exist in diatoms. One is a “biophysical CCM” in which CO_2_ and HCO_3_^-^ are transported by CAs and bicarbonate transporters. The other CCM is called “biochemical CCM” and involves a prefixation of inorganic carbon into C4 compounds as in C4 land plants, and then a conversion into C3 compounds and CO_2_ in proximity of RuBisCO favoring its carboxylation activity (Scarsini et al., 2019). Although the existence of a functional biochemical CCM was reported in *Conticribra weissflogii* (Grunow) Stachura-Suchoples & D.M. Williams) (formerly known as *Thalassiosira weissflogii*, there is no evidence for a functional evidence for a biochemical CCM in *P. tricornutum* (Haimovich-Dayan et al., 2013; Ewe et al., 2018).

The CA protein family is composed of proteins with different cellular sub-locations (e.g., chloroplast, PPS, cytosol) (Tachibana et al., 2011). Accordingly the up-regulation of genes coding proteins involved in the biophysical CCM was observed. Higher expression of “CCM genes” in phase 3 is consistent with the low CO_2_ available at this growth phase. It is interesting that the different gene sets were induced according to the growth light intensity.

In *P. tricornutum*, Tachibana et al. (2011) showed that putative CA1, CA2, CA6 but not CA7 are transcriptionally active and expressed independently of light and CO_2_ conditions, and thus appear to be synthesized constitutively. Similar results were found in our conditions. bCA4 and bCA5 were previously shown to be CO_2_ responsive and changes in mRNAs content are consistent with those reported previously by several authors under different growth conditions (Harada and Matsuda, 2005; Harada et al., 2005; Tachibana et al., 2011). Only for LL, the two bCAs (bCA4/PtCA1 and bCA5/PtCA2 (Tachibana et al., 2011), were strongly up-regulated in phase 3. Higher expression of these genes under LL in phase 3 may compensate the lower efficiency of RuBisCO, consistent with the decrease of mRNA expression in phase 3 LL for rbcS and rbcL, thus providing more CO_2_ around this enzyme. Also, down-regulation of the three HCO_3_^-^ transporters SLV (SLV4_1 SLV4_2 and SLV4_3) were observed in phase 3 under LL. Under LL, bCAs were mostly up-regulated whereas under HL, PPDK, SLVs and CAs were essentially over-expressed. Biochemical and biophysical CCM mechanisms together allow cells to increase inorganic carbon uptake and to keep high photosynthetic rates under low-CO_2_ environmental conditions. This action is required because of the progressive carbon shortage of the culture. Under HL and ML, the genes coding for the transformation of pyruvate to PEP within the biochemical CCM are up-regulated, generating CO_2_ that can be used for photosynthesis.

### Carbon Limitation Triggers the Reorientation of the Carbon Metabolism Toward Phospho*enol*pyruvate Formation

Regardless the photon flux density, the FbaC5 gene is highly up-regulated along the growth period and is accompanied by a significant down-regulation of TPI2 and GAPC1 both, involved in the reverse reaction that produce back trioses (Zgiby et al., 2000; Allen et al., 2011b). These changes may be interpreted as a lack of DHAP formation in the Calvin cycle. To feed the putative sink in DHAP, an import of this component from the cytosol using one of the triose phosphate translocators (TPT) can be postulated (for a review on transporters, see Marchand et al., 2018). The DHAP is probably transformed in Ru5P through the nonoxidative pentose-phosphate pathway or the Calvin-Benson cycle (Figure 6). The up-regulation of FBPC2 and FBPC4 genes under ML and HL, phase 3, suggests the use of the Calvin-Benson cycle for C5 regeneration along the growth period. The Ru5P can then serve as substrate for RuBisCO for binding CO_2_. In case of CO_2_ shortage (phase 3), Ru5P could be exported to the cytosol (Marchand et al., 2018) and metabolized through the oxidative pentose phosphate pathway after isomerization to D-xylulose-5-phosphaste (X5P) (Figure 6). The increase in the expression of the gene coding the cytosolic PGDH, the last enzyme of this pathway, agrees with this reasoning. Importantly, the reaction, from 6-phospho-D-gluconate (6PGN) to Ru5P catalyzed by PGDH, generates CO_2_ in the periplasmic space (PPS). This autogenerated CO_2_ can eventually be imported within the chloroplast and fixed by RuBisCO. Altogether, the data suggest that under CO_2_ shortage, part of the carbon pool circulates between the different cell compartments, limiting lipid accumulation even more. To summarize, the fixed CO_2_ is mostly used to form 3PG that is then transformed to 2PG. This conclusion is strengthened by the upregulation of genes coding for the enzyme catalyzing the transformation of 3PG to 2PG (PGAM) such as PGAM3 in phase 2 (>2-fold) under the three light intensities. 2PG is the substrate of enolase to form PEP, that, in turn, serves as a precursor of the Shikimate pathway or to produce pyruvate (Figure 6). The localization of PPDK, which converts pyruvate into PEP, is unclear. The sequence of the nonmaturated protein exhibits a plastid targeting presequence but the expression of PPDK::GFP fusion reveals a cytoplasmic localization (Ewe et al., 2018; Figure 6). Dual localization of proteins is an established possibility, including for PPDK in land plants (Parsley and Hibberd, 2006). According to Ewe et al. (2018), PEP and pyruvate generated in the cytoplasm could be reimported in the plastid by PEP and transporters, respectively. These transporteurs have still to be identified (Marchand et al., 2018). The gene coding the plastidic PPDK was among the highest up-regulated genes. Altogether, these results highlight that, regardless PPDK localization, the carbon flux is orientated toward PEP and pyruvate formation.

PEP can serve as acceptor for CO_2_/HCO_3_^-^ fixation by PEPC in the C_4_ route (Figure 6). The expression of PEPC1 (located in PPS) and PEPC2 (located in mitochondria) did not vary significantly as already observed in Valenzuela et al. (2012) in *P. tricornutum* under low CO_2_ conditions. However, the mitochondrial PYC1 and PEPCK, the enzymes allowing the conversion of pyruvate into PEP (through the fixation of HCO_3_^-^ and the formation of OAA), were also among the highest upregulated genes in phase 3, especially in ML and HL. This result suggests that under ML and HL, the carbon allocation in the mitochondria is also oriented toward PEP. Under LL, a significant part of OAA enters the TCA cycle for respiration as is suggested by the higher the respiratory activity under this light condition. Pyruvate can also be produced by the mitochondrial ME1 from malate. ME1 supplies both carbon and reducing equivalents in the form of NADPH for *de novo* fatty acid production (Kroth et al., 2008; Xue et al., 2015). Because no nitrogen depletion occurred in our conditions, it is unlikely that ME encoding genes stimulate lipid production (Yang et al., 2013; Xue et al., 2015). This would be consistent with the involvement of the mitochondrial pool of pyruvate in CCM to produce PEP that might be exported to other cell compartments, including plastids where it may serves as building blocks, e.g., aromatic amino acids and lipids. For aromatic amino acids, the expression of 6 genes coding enzymes involved in the Shikimate pathway were studied among which AroB and SK were slightly down-regulated in the three conditions, leading us to hypothesize that this is not the direction of carbon flux.

The activation of different pathways toward PEP (and pyruvate) synthesis highlighted at the mRNA level both in the plastid/cytoplasm and in the mitochondria is probably the consequence of a carbon limitation common in the different cultures along the growth period. Indeed, these two precursors constitute a hub from which the carbon is partitioned between the different biosynthetic pathways including protein and lipid biosynthesis. Also they are important intermediates in gluconeogenesis (the opposite direction of glycolysis) to produce energy (ATP), glucose and also storage (as chrysolaminarin) for the cell.

### The Carbon Partitioning Is Impacted by the Photon Flux Density

The PEP-pyruvate hub constitutes the starting point of different biosynthetic pathways including protein, lipid biosynthesis and aromatic compounds. Interestingly, under each light condition, the major compound was different in phase 1: under HL, proteins are proportionally higher whereas under ML and LL, chrysolaminarin and lipids, respectively, are proportionally higher. This is not unexpected because growth conditions under LL, phase 1, recall some conditions favoring lipid accumulation in diatoms: elevated amount of available C and reduced cell division rate (Nogueira et al., 2015). Under LL, the relative abundance of lipids decreased during the transition from phases 1 to 2 and then remained constant until phase 3 while the relative amount of chrysolaminarin increased. Indeed, mRNAs of the first enzyme of the chrysolaminarin synthesis, BGS1 (β-1,3-glucane glycosyltransferase) was found to be slightly overexpressed under LL compared to ML and HL and tend to decrease in phases 2 and 3. Under ML and HL, these conditions were only present transiently because division rate increased rapidly after the start of culturing. To sustain this growth, most of the C is incorporated into simple sugars that are used to generate ATP through respiration, which strongly increased during the phases 1–2 transition. The ATP produced could be imported in the chloroplast using NTT transporters (Ast et al., 2009) and used, for instance, in the Calvin cycle. Out of the three FbaC genes coding plastidial Fba (Allen et al., 2011a), only FbaC5, that represented *circa* 30% of the total “plastidial” FbaC mRNA (data not shown), was up-regulated in all three conditions. Indeed, in the land plant *Arabidopsis thaliana*, FBA is also one of the three Calvin-Benson cycle enzymes that was most sensitive to environmental perturbations and found to have an important rate-limiting role in regulating the carbon assimilation flux (Sun et al., 2003). Interestingly, no particular regulation of the genes coding the RuBisCO subunits was observed except under LL for which a reduction was observed along the growth period, confirming earlier reports (Kroth et al., 2008).

As a response to HL, lipids accumulated during phase the 2-to-phase 3 transition, as reported by Valenzuela et al. (2012), Mus et al. (2013), and Wu et al. (2015) but here, the accumulation was limited because of the progressive depletion in C. The origin of the lipid biosynthesis, i.e., PEP or pyruvate, is still under debate. Some authors have assumed that lipid biosynthesis branches from PEP (Kroth et al., 2008; Mus et al., 2013), while others noted that it goes through pyruvate (Radakovits et al., 2012; Yang et al., 2013; Ge et al., 2014; Schwender et al., 2014).

The pyruvate dehydrogenase complex (PDC) is an important enzyme of lipid metabolism. Three isoforms of pyruvate dehydrogenase, namely PDH1, PDHB1, and PDHA1, were found in the *P. tricornutum* genome (Chauton et al., 2013). PDH1 gene expression decreased in phases 2 and 3 while the expression of the PDHA1 increased. The PDHB1 level did not change significantly. Moreover, the mRNA expression of the two isoforms of acetyl-CoA carboxylase (ACC1 and ACC2), which catalyze the conversion of acetyl-CoA into malonyl-CoA for fatty acid production, indicated a singular pattern: a higher expression of both genes in LL and an up-regulation of ACC2 in phase 3, though not significantly under HL. It seems that a higher expression of these genes during phase 3 under LL speeds up the conversion of pyruvate to malonyl-CoA. This compound is a key cofactor of the fatty acid biosynthesis.

### The Involvement of the Photorespiration Pathway Is Not Enhanced, Even Under HL

It is well recognized that O_2_ and CO_2_ are in competition for the RuBisCO active site. When the gas partial pressure surrounding RuBisCO is in favor of O_2_, RuBP enters the photorespiration pathway of which the first step consists in the oxidation of RuBP to 2PGL by RuBisCO (Figure 6; Sage and Stata, 2015). Therefore, in the case of C limitation, it could be expected that photorespiration will be strongly activated. Besides its role in diverting carbon atoms, photorespiration plays a critical role in nitrogen metabolism in diatoms and a role in excess energy dissipation under stress conditions (Kroth et al., 2008). This is unlikely in our conditions because qN measurements suggest that the capacity to dissipate the excess of absorbed light energy was too low to be saturated. The gene coding PGP, the enzyme catalyzing the formation of glycolate from 2PGL was down-regulated in LL, ML, and HL in phases 2 and 3 compared to phase 1. These changes were accompanied by the down-regulation of the expression of two other photorespiratory genes coding the mitochondrial GOX2 and GDCP. The former role was probably more easily filled as the N in the medium remained high whereas the latter is unlikely as the capacity to dissipate the excess of energy through the nonphotochemical quenching was far from saturation. Altogether, our results highlight that photorespiration is not particularly enhanced, even under HL.

## Conclusion

To conclude, our results show that the impact of light intensity on cell development, physiology and gene regulation of *P. tricornutum* depends on growth phase, i.e., the cell’s physiological state. Generally, diatom cells adapted to different light conditions in efficient ways to keep cell growth, processes and regulation. In all light conditions C-deficiency was mostly responsible for the occurrence of the plateau phase in these cultures, whereas no deficiency of N was observed in the cultures. A common point in all growth photon flux densities was the modification of gene transcription that would allow the synthesis of PEP and, in some cases in the reverse direction, to pyruvate. Our results confirm the recent finding of the existence of a pyruvate hub in microalgae (Smith et al., 2012; Heydarizadeh et al., 2017) and also show that reorientation of carbon metabolism might occur according to combination of environmental factors. Altogether, the data set presented in this manuscript shows that even in conditions of carbon deprivation, diatoms orient their metabolism toward the production of lipids.

## Author Contributions

PH, JM, and BS conceived the idea of the manuscript. PH, BV, BH, EL, GW-C, AC-M, GB, JM, and BS performed the experiments and wrote the related parts of the manuscript. PH, JM, and BS wrote the introduction, the discussion and the conclusions as well as prepared the figures.

## Conflict of Interest Statement

The authors declare that the research was conducted in the absence of any commercial or financial relationships that could be construed as a potential conflict of interest.

## Footnotes

1 http://www.curveexpert.net/
