# Supplementary material for: Carbon Orientation in the Diatom Phaeodactylum tricornutum: The Effects of Carbon Limitation and Photon Flux Density
Source: Front Plant Sci. 2019 Apr 16;10:471. doi: 10.3389/fpls.2019.00471 (PMC6477932; doi:10.3389/fpls.2019.00471)
Supplement: Supplementary file 1 [file Table_1.DOCX]

## Supplemental Data 1: List of enzymes and related genes

Table SD1. Name and sequences of the primers used, ID and localization List of the enzymes and of their corresponding gene/isogene(s), protein ID (http://genome.jgi.doe.gov/Phatr2/Phatr2.home.html) and primers.

|  | Enzyme | Gene abreviation | Protein ID | Compartment | Primer-F (5'-3') | Primer-R (5'-3') |
| --- | --- | --- | --- | --- | --- | --- |
| 1 | Pyruvate phosphate dikinase | PPDK | 21988 | Plastid/cytosol | CAGCGGGTTGAGCAAGACAT | TCATACCAGGCATGGAAATGG |
| 2 | Phospho*enol*pyruvate carboxylase | PEPC1 | 51136 | plastid | AATTGCCCAAGCTGGGTTTC | ATGACGTAGGCTCCCAAACAA |
|  |  | PEPC2 | 27976 | mitochondria | GCGACTCACATCAAGCATCAG | GTCTTCGCCGCAAGGAAAC |
| 3 | Malate dehydrogenase | MDH | 51297 | mitochondria | CAACCGCTCTCGATGCTTCT | TGTGACTGAGATCCGCAGCTA |
| 4 | Malate synthase | MS | 54478 | peroxisome | GGGCGGTATGAGTGCATCA | AGCTGTCACTTCGCGCAACT |
| 5 | Phosphoenolpyruvate carboxykinase | PEPCK | 55018 | mitochondria | GCCGAATACGGTGACACATTT | CTTCGGTCTCGGATCCCTTAT |
| 6 | Phosphoglycolate phosphatase | PGP | 48026 | plastid | ACCTGGCACCAATCATTTGG | CGGTAAACCACACCGTCACA |
| 7 | Pyruvate carboxylase | PYC1 | 30519 | mitochondria | TTTCGACCGCTGGCATTTAT | GCGACTGGAGACTTGCTGGTA |
|  |  | PYC2 | 49339 | plastid | GGAAAATCCCGAGCGAGACT | GGAGTATCCAATGCCGTCCAT |
| 8 | Sedoheptulose-1,7-biphosphatase | SBP | 56467 | cytosol | GGAGCGGCAGGAGGATTAC | GGCGAGAGCTTCGTACGATT |
| 9 | Hydroxypyruvate reductase/glycerate | HPR | 56499 | mitochondria | GATCTCCGGCCTCGTGATT | GGAGGCTTCTCGGACTGAAA |
| 10 | Glyceraldehyde-3-phosphate dehydrogenase | GAPC1 | 22122 | plastid | AGCCGACTACGTCTGCGAAT | GGGTGCCGAGTAGATGACCTT |
|  |  | GAPDH | 23598 | cytosol | TGTGAGTCCGCTGCCTATCTC | CGTCGACGGTAAAGGATTGG |
|  |  | GapC4 (TPI/GapC3) | 32747/25308 | mitochondria | TCCTCCATGGACGTGGTTTC | TGAGACCCTCCTTGAGTCCAA |
| 11 | 6-phosphogluconate dehydrogenase | 6PGDH | 45333 | cytosol | CTCAGCAAGCCTCGGAAAGT | AATTCGCTGATCGCTTCGAT |
| 12 | Oxoglutarate/malate transporter | OMT1 | 8990 | mitochondria | ATTACCTCAATCAACGCCGAAA | GGCGAGCGCATAGATGAGA |
| 13 | Pyruvate kinase | PK1 | 22404 | plastid | CGGATGTGTTGGCAAAGACA | AACCGCTGCCACTGTTGTTT |
|  |  | PK2 | 49098 | cytosol | GTAACGGCCACACAGATGCTT | AGTCCGTGCCGTCCAAGAC |
|  |  | PK3 | 56445 | cytosol | ATTGAATCGGGCATGAATGTG | TGTTTTGTGCAGCCTGACGTA |
|  |  | PK4a/PK4b | 45997/27502 | cytosol | ATTTCGTCCGGGCTTGAGT | TGCCCAACAGCAATCCACTA |
|  |  | PK5 | 49002 | mitochondria | TGCAGCTACACCGTTCTATAATTCC | CGTACGGCTTCAGCTCTCAGA |
|  |  | PK6 | 56172 | mitochondria | CCTCCACTCGTCTCGGCATA | TGCAACTGTCGGGCTAACTTG |
| 14 | Bicarbonate transporter | SLV4_1 | 45656 | plastid | GCCCAGCCGTTGACAATC | CAGCATCTTCGCCAATTGTG |
|  |  | SLV4_2 | 32359 | plastid | CGGTGCTCTGGATCGGAATA | ACCAAGTGCAAGGATGATGGTT |
|  |  | SLV4_3 | 54405 | membrane | TGGTTGCCTCCATTCTGATCT | TGAGTCGGCGTGATGCTATC |
| 15 | Triose phosphate isomerase | TPI_1 | 18228 | plastid | ACCTGCCGTCAGATTTTGGT | CTGACGCTTCCTCCGTAAAGA |
|  |  | TPI_2 | 50738 | plastid | GGGTAGTCTTTGGCGAAACG | GATTCGGATTCGCCAATGC |
| 16 | Fructose-1,6-bisphosphatase | FBP | 23247 | cytosol | GCGATTGCGACCTCCTGTAA | CGTCGCCGGTTGAGTTG |
|  |  | FBPC1 | 42886 | plastid | ACATTGGGCTCTGGAGTCGAT | AAATGGGTCCAGAGGAAGGAA |
|  |  | FBPC2 | 42456 | plastid | GTGACGCTGACGCGCTTTA | TTGCCTTACAGGCCGTATCG |
|  |  | FBPC3 | 31451 | plastid | TTTGGGTATCCTGGCGACAA | ACAATCCACCCGCCTGTTC |
|  |  | FBPC4 | 54279 | plastid | AGCACCCGCTGTCGATTC | AAACCGGCTCAACGTAATGG |
| 17 | Glucose-phosphate isomerase | GPI_3 | 56512 | plastid | CCACGCAACGGTCAATCTT | CGAAACGAGCTGTGGTCGAT |
|  |  | GPI_1 | 23924 | cytosol | TCAGCAAGGCGACATGGAA | GGTGCCAGGTTCACCGAATA |
|  |  | GPI | 53878 | cytosol | AGCGCAGCAGCACATTCAT | CGGGCTGTGCAAAAAAGTTT |
| 18 | Fructose-bisphosphatase aldolase | Fba3 | 29014 | cytosol | CGGATCGGTCGCTTTGG | AGTGGTCGGAATGAAGGATGA |
|  |  | Fba4 | 42447 | cytosol | GGACGAGAATCATTACGAGTAAAGC | GCGGTCCATCGTATCTTCAAA |
|  |  | FbaC1 | 825 | plastid | TGGTTGCCCTGGTTTGATG | CTTCGGAGAGATCCAACATGTG |
|  |  | FbaC2 | 22993 | plastid | ACCGGACAAGCCCAACAAG | TAGGTCGGCGTACGATTTGC |
|  |  | FbaC5 | 51289 | plastid | TGACGTTCTGACCGATGCA | GCCAGAGCACCAGGTTTCAC |
| 19 | Carbonic anhydrase | aCA1 | 35370 | secreted | GACACTTCCGGTACTATTTGGAGAA | GTCGCTGTAGTTGGCATCGA |
|  |  | aCA2 | 44526 | secreted | CATACCATTGACGGTTTCAACACT | AGGTTGCGACTTTTTTGACGAA |
|  |  | aCA3 | 55029 | secreted | CGCTTACAGTTCCAGGTGTATCG | TCGGCTCCCGAGAAATTG |
|  |  | aCA6 | 54251 | secreted | TCGCAGTGGTGGTTCCTTTC | CATATGCAATTCGCCATCGT |
|  |  | aCA7 | 42574 | secreted | AGCAAAGGTTTCCCGGATTG | GTGTGACTTCGGCGTCGTATC |
|  |  | bCA4 (PtCA1) | 42406 | plastid | ACGTTATCGTCTGTGGTCACTATGA | CGGATGTTACGGAGCCAGAT |
|  |  | bCA5 (PtCA2) | 45443 | secreted | CGTCGATTTGAACGTCATTGA | CCGCCATCCTTGTAGCTTTC |
| 20 | Phosphoglycerate mutase | PGAM_1 | 42857 | plastid | CCGTCTGAGAACGAGGTTGTG | CGGGTACGCTGCCCATAC |
|  |  | PGAM_2 | 43253 | mitochondria | CAAGACAGTTATGGTGGCAGATTC | CCAAGGCATCCTCCATCTTC |
|  |  | PGAM_3 | 43812 | plastid | GATTGCGACCACCAGAAAGG | GTTGACGTGCCGAAAAAGGA |
|  |  | PGAM_4 | 51298 | plastid | AGGCGTTCCGCTCGTTTAC | GTCCTGATAACGGCGCAATC |
|  |  | PGAM_5 | 26201 | mitochondria | AAGCCTGCCGATTCCTCTCT | TTGCATGCTTGCACAGAATTT |
|  |  | PGAM_6 | 33839 | mitochondria | CCGCGGTACAATGGCTTT | CAAAATGGTACAACGCGTTCA |
|  |  | PGAM_7 | 35164 | mitochondria | CTGGACCAACGTCGGTCTTT | CGATCGAGTACCCGTGCAA |
| 21 | Glycine decarboxylase | GDCP | 22187 | mitochondria | CGTATTGCTTTGGAGGATTCG | GGACGATCCCACTTCTCATTG |
|  |  | GDCT_1 | 56477 | mitochondria | TCGAAACGCGAAGCAATCT | GGGAGCGTACCAGGATGGA |
|  |  | GDCH | 32847 | mitochondria | TTCATTTGACAAAGGCGATAGC | TCGACGACAGTTCCAGAGATTG |
| 22 | NAD Malic enzyme | ME1 | 56501 | mitochondria | TCGTATTATGAACATCCATTTGCA | TCGAGCATGACGTTCTTACGAA |
| 23 | Glycolate dehydrogenase/glycolate oxidase | GOX1 | 22568 | peroxisome | GATGCCTTTGCTCGCTGGTA | CCGGCATACTGAGTCCTTGTC |
|  |  | GOX2 | 50804 | mitochondria | CAAGGACGCGTATTCCGAAT | TGTGACGTCCTGGCCAAAG |
| 24 | RubisCO small subunit | rbcS | ABK20640 | plastid | TGCTTACGGTACTGAAAGTTGTGTT | AAGCAATACGACGACCTTCAAGT |
|  | RubisCO large subunit | rbcL | AAF07200 | plastid | TTGGGCGCGTGATAATGA | AAATAACACGGAAGTTGATACCATGA |
| 25 | 3-deoxy-7-phosphoheptulonate synthase | AroA | 24353 | plastid | CACGCCTGGAATTTGGACTT | GAAACGGAGCGACTCGTCAA |
| 26 | 3-dehydroquinate synthase | AroB | 20809 | plastid | CTTTGAGTGGCAGGAGGATCA | AAGATCGCGTAATGGCGAAA |
| 27 | Shikimate/quinate 5-dehydrogenase /  3-dehydroquinate dehydratase | AT3G* | 45535 | cytosol | GGCAGAGTCCCGTTATTCGA | CGCGCATTGCTGAAACAAT |
| 28 | Shikimate kinase | SK* | 49363 | plastid | ATCGAAAGAGCAACAGGAATGAC | GTCCAAAACTTGCGCTTCCA |
| 29 | 3-phosphoshikimate 1-carboxyvinyltransferase | EPSP* | 18246 | plastid | TCCCTGGTTCCAAATCTCTGA | CAAATGTCGTCGGAATCCAA |
| 30 | chorismate synthase | AT1G* | 43429 | plastid | TTGAGAGTGGAGACGGCTTTG | GGTCTCGGGATCCACGTAGA |
| 31 | Acetyl-CoA carboxylase | ACC1 | 54926 |  | CATGTCCGAGGCGATTGG | TTACTGTTCGCACACCCATAGC |
|  |  | ACC2 |  |  | ACGGAAGCACATCCAATAACG | TCTCCGGCAGCAAAATGTATC |
| 32 | Pyruvate dehydrogenase | PDH1 | 20360 |  | CAGGTTCACACTTACCGCTTCA | CGCCCACGCCTTTTCTT |
|  |  | PDHA1 | 55035 | mitochondrial precursor | TGGCGGGCTTGATTCTGA | CGGCTCGTCGTTCTCACA |
|  |  | PDHB | 20183 | mitochondrial precursor | ACGCGACCATTATCTCCTTCA | TTCCACGGATACGCCTTTTT |
| 33 | Glycosyl transferase | BGS1 | 55327 |  | CCTTTGCCAACGACCATTCT | GGTGACCATAGTGGAGACGGATA |
