# Supplementary material for: Carbon Orientation in the Diatom Phaeodactylum tricornutum: The Effects of Carbon Limitation and Photon Flux Density
Source: Front Plant Sci. 2019 Apr 16;10:471. doi: 10.3389/fpls.2019.00471 (PMC6477932; doi:10.3389/fpls.2019.00471)
Supplement: Supplementary file 2 [file Table_2.DOCX]

## Supplemental Data 2: Cellular pigment quota


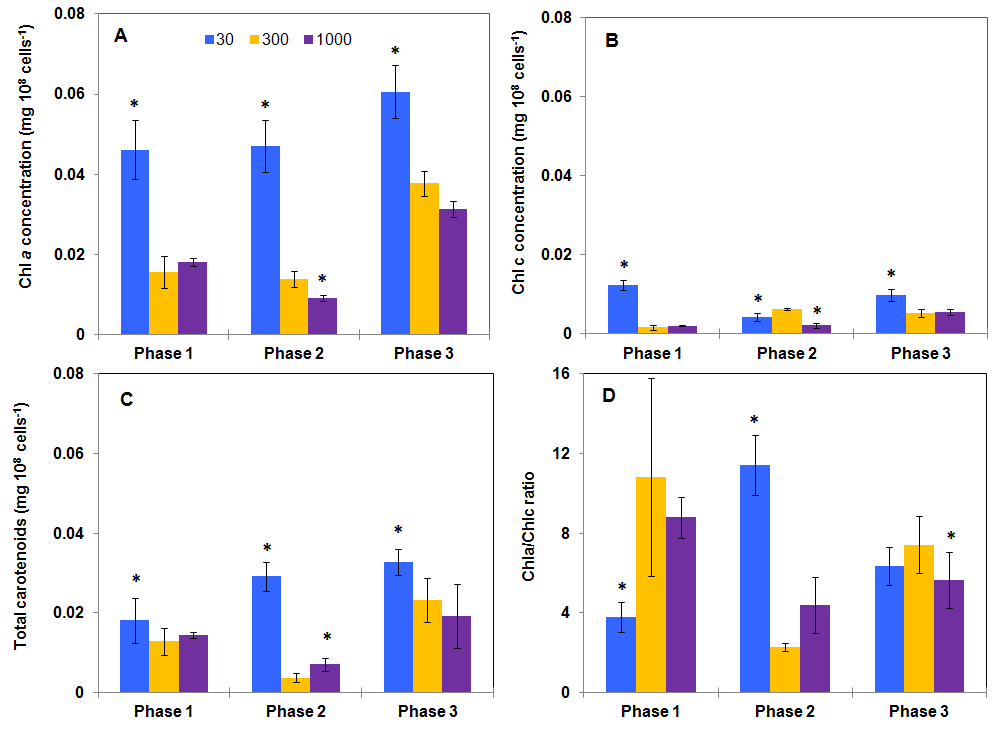


Figure SD2.1. Pigments content per cell in *Phaeodactylum tricornutum* grown under different light intensities.

Changing photon flux densities altered pigment concentrations. The time-course of Chl *a* and total carotenoid accumulations were very similar under ML and HL: both decreased during the transition between phase 1 to phase 2 and significantly increased in phase 3. Under LL, the level of individual pigments increased from phase 1 to phase 3 except Chl c that first decreased and then increased. During phase 1 and phase 3, Chl c content was higher under LL than under ML or HL. Under LL, the ratio increased from phase 1 to phase 2 and then decreased until phase 3 is reached. At the end of phase 3 the ratio was similar for all conditions. Interestingly, carotenoids mostly followed the Chl *a*/Chl c ratio except during phase 2 to phase 3 transition under LL.

Data are mean values ± SE (n = 3) and error bars represent SD. Means followed by asterisks are significantly different from the corresponding value for ML (*p* ˂ 0.05).
