# Supplementary material for: Carbon Orientation in the Diatom Phaeodactylum tricornutum: The Effects of Carbon Limitation and Photon Flux Density
Source: Front Plant Sci. 2019 Apr 16;10:471. doi: 10.3389/fpls.2019.00471 (PMC6477932; doi:10.3389/fpls.2019.00471)
Supplement: Supplementary file 3 [file Table_3.DOCX]

## Supplemental Data 3: Kinetic of chlorophyll *a* fluorescence - Management of the incoming light energy.

Figure SD3 displays Chl fluorescence induction kinetics of *P. tricornutum* cells recorded under different light conditions. Comparison of the curves clearly indicated the differences between Fs levels The F_0,_ F_M,_ F’_0,_ F’_M,_ F”_0_ and F”_M_ were used to quantify parameters (Table SD3.1) describing how the photosynthetic apparatus was managing the absorbed energy. They are briefly described below. For a comprehensive description, the reader is referred to Roháček et al. (2008, 2014).


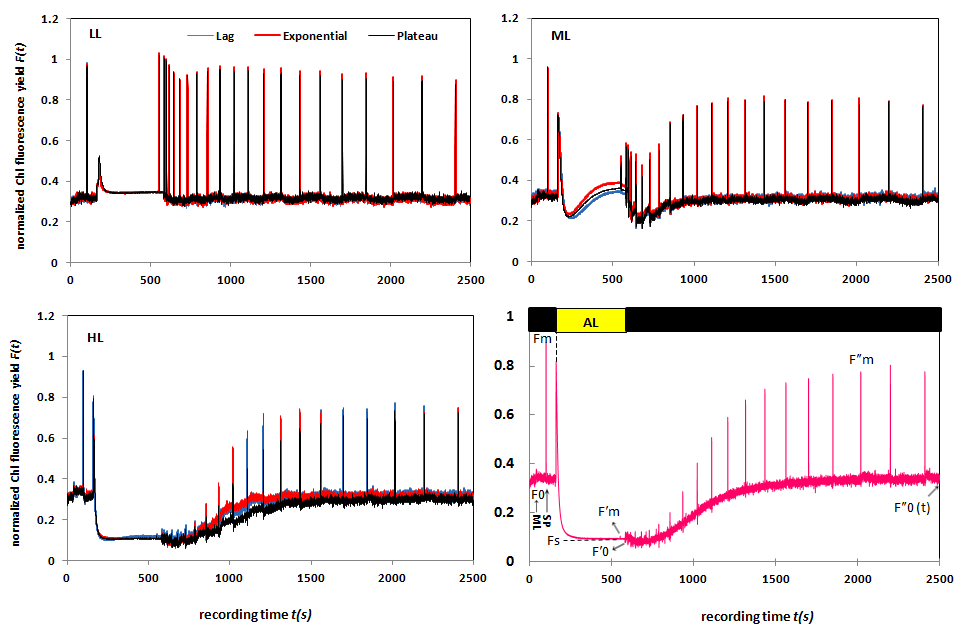


|  | Figure SD3.1. Chl fluorescence induction kinetics of *P. tricornutum* under LL, ML and HL. F_0_: minimal fluorescence yield of dark-adapted sample with all PS II centers open; F_M_: maximal fluorescence yield of dark-adapted sample with all PS II centers closed; F'_M_: maximal fluorescence yield of illuminated sample with all PS II centers closed; F'_0_: minimal fluorescence yield of illuminated sample with all PS II centers open (measured immediately after acclimation to light); AL: actinic light; ML: modulated light; SP: saturation pulse serving for transient full closure of PS II centers. |
| --- | --- |
|  | Table SD3.1. Commonly fluorescence parameters used throughout the text for quantification of the relative electron transport rate (rETR) as well as the photochemical (Φ_P0_, qP, Φ_P_, Φ_II_) and nonphotochemical (qN, q0, NPQ) processes (Equations according to Roháček et al., 2008). |

| *Photochemical quenching parameters* | Formula |
| --- | --- |
| rETR Relative electron transport rate | E (F′_M_-F_S_)/F′_M_ |
| Φ_P0_ Maximum quantum yield of PSII | F_V_/F_M_ = (F_M_-F_o_)/F_M_ |
| Φ_II_ Photochemical efficiency of PSII | (F′_M_-F_S_)/F′_M_ |
| qP Photochemical quenching | (F′_M_-F_S_)/(F′_M_-F′_o_) |
| 1-qP Degree of PSII reaction centre closure | (F_S_-F′_o_)/(F′_M_-F′_o_) |
| *Non-photochemical quenching parameters*: |  |
| NPQ Non-photochemical quenching | (F_M_-F′_M_)/F′_M_ |
| q_0_ Relative change of minimum Chlorophyll Fluorescence | F_o_-F′_o_/F_o_ |
| q_N_ Non-photochemical quenching of variable Chlorophyll Fluorescence | F_V_-F′_V_/F_V_ |

*Maximum quantum yield of PSII photochemistry* (Φ_P0_): it quantifies the maximum photochemical efficiency of PSII in a dark-adapted state and is serve as a proxy of the fitness of the photosynthetic apparatus.

*Effective quantum yield of photochemical energy conversion in PSII* (ΦII): it quantifies efficiency of photochemical processes during conversion of the excitation energy by actually open PSII reaction centers.

*Photochemical quenching of variable Chlorophyll fluorescence* (qP): it quantifies the actual photochemical capacity of PSII and is proportional to the fraction of PSII reaction centers being actually in the open state under actinic irradiation.

*Degree of PSII reaction centre closure* (1-qP): it quantifies the proportion of centers that are closed and sometimes termed to “excitation pressure” on PSII (Maxwell and Johnson, 2000).

*Nonphotochemical chlorophyll fluorescence quenching* (NPQ, qN): it reflects the excess radiation converted to heat during the actinic irradiation. Its extent correlates mostly to diatoxanthin formation. NPQ calculation differs from that of qN in the fact that the former relies on maximum fluorescence levels whereas the later relies on the variable fluorescence (Roháček et al., 2008).

*Relative change of minimum Chlorophyll fluorescence* (q0): it is linked to processes of the nonphotochemical nature activated in thylakoid membranes under the actinic irradiation.

qN analyses were performed as explained in Roháček et al. (2014). From the mechanism point of view, qNi relies on the dissipation of the proton gradient (∆pH relaxation) and diatoxanthin epoxidation. qNf seems to be related to a fast conformational changes occurring within the thylakoid membranes in the vicinity of the PSII complexes, whereas qNs could be related to photoinhibition and/or partial dissipation of the pH gradient (Roháček *et al.,* 2014).

Table SD3.2. Variations of the relative electron transfer rate according to the phase and photon flux densities.

|  | LL | ML | HL |
| --- | --- | --- | --- |
| Phase 1 | 20.22 | 134.30 | 193.73 |
| Phase 2 | 20.03 | 135.48 | 177.32 |
| Phase 3 | 19.27 | 74.22 | 89.22 |

Literature

Roháček, K., Soukupová, J., Barták, M. Chlorophyll fluorescence: A wonderful tool to study plant physiology and plant stress. In *Plant Cell Compartments - Selected Topics.* Schoefs, B., Ed.; Research Signpost: Kerala, India, 2008; p 41

Roháček, K., Bertrand, M., Moreau, B., Jacquette, J., Caplat, C., Morant-Manceau, A., Schoefs, B. (2014) Relaxation of the non-photochemical Chlorophyll fluorescence quenching in diatoms: kinetics, components and mechanisms. *Phil. Trans. R. Soc*. 369, 1640-20130241.
